# The Influence of Identity Within-Person and Between Behaviours: A 12-Week Repeated Measures Study

**DOI:** 10.3390/bs15050623

**Published:** 2025-05-03

**Authors:** Kristie-Lee R. Alfrey, Matthew Condie, Amanda L. Rebar

**Affiliations:** 1 Motivation of Health Behaviours Lab, Appleton Institute, CQUniversity, Bundaberg, QLD 4670, Australia; 2 Motivation of Health Behaviours Lab and Cluster for Resilience & Well-Being, Appleton Institute, CQUniversity, Adelaide, SA 5034, Australia; m.condie@cqu.edu.au; 3 Motivation of Health Behaviors Lab, Department of Health Promotion, Education, and Behavior, Arnold School of Public Health, University of South Carolina, Columbia, SC 29201, USA; arebar@mailbox.sc.edu; 4 Motivation of Health Behaviours Lab, Appleton Institute, CQUniversity, Rockhampton, QLD 4701, Australia

**Keywords:** identity theory, behaviour change, behavioural intention, self-determined behaviour, habit

## Abstract

People act in ways that align with the values and roles that constitute their identity. However, the consistency of identity’s influence across different behaviours, and whether identity influences behaviours directly or indirectly via intention, self-determined motivation, or habit, remains uncertain. Participants (*N* = 98; *Mage* = 30.4 years, *SD* = 11.7 years) completed up to 12 weekly surveys, self-reporting engagement in physical activity, student, and support-seeking behaviours, and behaviour-associated identity, intention strength, self-determined motivation, and habit. Stepwise multilevel models tested the between- and within-person associations of identity with behaviour, and whether the relationships remained after accounting for intention, self-determined motivation, and habit. Results suggested identity as stable, with the most variability at the between-person level. Identity was associated with behaviour at both within- and between-person levels, with the exception that support seeking and identity were only associated between-person. For student behaviour and physical activity, the identity–behaviour relationship at the within-person level waned and became non-significant after accounting for intention, but not self-determined motivation or habit. These findings highlight that identity may be difficult to change. However, as identity is associated with a range of behaviours, a person’s identification with a particular behaviour may be valuable for tailoring behaviour change interventions, specifically through or in the same way as behavioural intentions.

## 1. Introduction

A person’s identity is constructed through the meanings they derive from being a person—that is, the social roles and memberships a person experiences across their lifetime informs the values and beliefs they internalise, which are then reciprocally supported by the social contexts the person associates with (Burke, 1980; Stets & Burke, 2000; Stryker, 1968). For example, if a person grows up in a family that values physical activity (social context), that person may internalise those values as their own (self identity) and engage in activities and communities that encourage physical fitness (social identity). Identity theory (Burke & Stets, 2009) suggests that people behave in ways that reflect their personally and socially held values and beliefs. A body of empirical research spanning a range of behavioural outcomes supports this notion that people engage in, and commit to, behaviours that reflect their identity (Alfrey et al., 2023; Berkman et al., 2017; Rhodes et al., 2022). While evidence supports that identity is a likely determinant of behaviour, the construct of identity has not commonly been integrated into behavioural interventions (Tarrant et al., 2020). This lack of application may potentially be due to the uncertainty of the process by which identity influences behaviour. For example, it is unclear whether identity is a direct, independent determinant of behaviour, or as an indirect influence working through other motivational determinants such as intention, motivation or habit. Amidst investigations connecting identity to behaviour, the question of precisely how identity influences behaviour, and if it influences various behaviour types differently, is of importance (Alfrey et al., 2023; Barnett et al., 2021; Rhodes, 2017).

### 1.1. The Influence of Identity on Behaviour Change Mechanisms

Behavioural intention, self-determined motivation, and habit are key determinants of effective and long-term behaviour change. The Rubicon model of action phases (Heckhausen & Gollwitzer, 1987; Heckhausen, 2007) describes behavioural intention as being formed when the decision to intend to engage in a behaviour is made (pre-decisional motivation) and is then followed by strengthening of intention through planning (pre-actional volition). Self-determined motivation is then suggested as a key factor during the action initiation stage (actional volition) and the behavioural maintenance stage (post-actional motivation), to manage perceptions of personal control (autonomy) and satisfaction (enjoyment and outcomes). Habit formation is then theorised to support long-term engagement in the behaviour through the non-consciously associated context-cued impulse to enact the behaviour (Gardner & Rebar, 2019; Gardner et al., 2024).

The conceptualisation of identity (as per Burke & Stets, 2009; Stets et al., 2020) has commonalities with other determinants of behaviour, including intention, self-determined motivation, and habit For example, self-determination theory highlights the links between identity and self-determined motivation as being associated with identified regulation, intrinsic enjoyment and internalised rewards gained from engaging in a desired behaviour (Ryan & Deci, 2000; Ryan & Deci, 2003). However, of the limited behavioural research evidencing how identity influences the relationship between self-determined motivation and behaviour, results are mixed. Most behaviour research in this space infers indirect associations between identity and self-determined motivation (García et al., 2023; Kindelberger et al., 2020; Murnieks et al., 2014), while fewer others infer a more direct association between identity and self-determined motivation (Hwang, 2010).

Contrasting the limited evidence pertaining to identity and self-determined motivation is the depth of evidence pertaining to the relationship between identity and behavioural intention. For example, identity has been a regular feature within research focused on intention-based theories, such as the Theory of Planned Behaviour (Ajzen, 1985, 1991). Much of this research generally supports the notion that identity is an independent influence working alongside intention to influence behavioural outcomes (Buhrmester et al., 2015; Nigbur et al., 2010; Smith et al., 2008; Sparks & Shepherd, 1992). However, there is also some evidence that suggests identity may predict intentions but not behaviour (Gardner et al., 2012b; White et al., 2008), inferring that identity may influence behaviour indirectly through intentions, by serving as a value that is reflected upon when intentions are generated (Ajzen, 1991; Berry et al., 2014; Fielding et al., 2008; Fishbein & Ajzen, 2010; Gardner & Lally, 2013; Hagger & Chatzisarantis, 2006).

In contrast to intentional influences on behaviour, there is also emerging evidence to suggest that the influence of identity on behaviour may be processed automatically and outside of conscious awareness (Michie et al., 2011; Rhodes, 2017; Rhodes et al., 2023). That is, the influence of identity on behaviour may not solely rely on deliberate, conscious reflection represented by intention formation, but rather through automated processes aligned with those described by habit theorists (Gardner & Rebar, 2019; Rhodes et al., 2023). Research considering identity and habit suggests the two are conceptually different and influence behaviour in different ways (Gardner et al., 2012b). However, research also suggests that when habitual behaviour relates to a person’s identity, the identity–behaviour link becomes mutually reinforcing (Hamilton et al., 2023), is more readily identified and internalised (Rhodes et al., 2016), self-esteem is improved, and the desire to achieve the ideal self is heightened (Verplanken & Sui, 2019). In all, evidence relating to if and how identity relates to habit, intention, and self-determined motivation to influence behaviour remains mixed.

### 1.2. The Influence of Identity Across Different Behaviours

The identity–behaviour relationship has been researched across a wide variety of behaviours. For example, research has connected identity with health behaviours (Cushan-Kain et al., 2022; Forshaw et al., 2023; Gardner et al., 2012b; Rhodes et al., 2016), student behaviours (Chorba et al., 2012; Hardy et al., 2013), occupational and professional behaviours (Cardoso et al., 2014; Maxwell & Visek, 2009), and pro-social behaviours (Udall et al., 2020, 2021). Together, this research suggests that people who identify with a behaviour tend to engage in that behaviour more, compared to those who do not identify with the behaviour. Contrastingly, some research has reported relatively weak to no association between identity and behaviours such as aggression (Maxwell & Visek, 2009), sexual risk-taking (Hardy et al., 2013), travel mode behaviours (Murtagh et al., 2012), and moral behaviours (Hertz & Krettenauer, 2016). Across this body of evidence, there is a wide variety in the magnitude of association between identity and behaviour types (Alfrey et al., 2023), thus creating uncertainty in terms of if and how identity influences different types of behaviours.

Inconsistencies in methods between studies disallow for clear, controlled testing of the interrelations of identity and behaviour alongside other determinants. For example, as exampled across existing reviews (Alfrey et al., 2023; Cardoso et al., 2014; Udall et al., 2020, 2021), studies of identity and behaviour vary in terms of the conceptualisation and measurement of variables (e.g., dichotomous responses vs continues measures, self-report vs implicit measures vs device data), population differences (e.g., culture, gender, age), and individual differences (e.g., past behaviour, level of access to behaviour-supporting resources such as time, equipment, finances). A single study, in which identity is conceptualised and measured consistently across multiple behavioural outcomes, is needed to establish if the differences between existing studies are a result of the variation in variables and behavioural outcomes or not.

Identity is typically considered a relatively stable concept, with low levels of change over time (Chen et al., 2023). However, identity is founded in symbolic interactionism (Mead, 1934) and informed by the subjective interpretation of social norms and expectations; thereby, the consideration of dynamic between-person effects may be warranted when studying identity and behaviour. Contrastingly, intention, self-determined motivation, and habit have been evidenced to fluctuate across time (Alfrey et al., 2024; Gardner & Lally, 2018; Lally et al., 2010; Lavigne et al., 2009; Ram et al., 2014; Rebar et al., 2018; Shiffman et al., 2008), suggesting dynamic within-person effects may also be important factors in the study of the identity–behaviour relationship. Research that considers the identity–behaviour relationship consistently across various behaviours, while acknowledging dynamic within- and between-person effects, is needed to contribute to evidence on whether or not identity might influence a range of behaviours in the same way.

### 1.3. The Present Study

The present study aims to investigate the influence of identity consistently across three distinct behaviours: physical activity, student behaviour, and support seeking. These behaviours were chosen as each was considered to emphasise different motivational factors. For example, physical activity often highlights autonomous regulation (Manninen et al., 2022; Teixeira et al., 2012), student behaviour tends to focus on achievement goals (Urhahne & Wijnia, 2023), and support seeking primarily underscores the importance of social connection and may also be influenced by cultural values and stigma (Szkody et al., 2024). It was considered that any differences in identity–behaviour associations, across the three behaviours, may be, at least in part, attributed to these underlying motivational factors. The associations will be tested at both the between- and within-person levels. Additionally, it will be tested if the associations between identity and behaviour are beyond those of intentions, self-determined motivation, and habit. Simultaneous study of these variables and behaviours within the one study provides conceptual and measurement consistency previously investigated independently; thereby offering a level of outcome consensus that has not yet otherwise been offered. A repeated measures approach will provide insights into between-person differences as well as potential within-person fluctuations of variables and their interrelations across various points in time and context (Lindsey & Lambert, 1998; Shiffman et al., 2008). Given the theoretical premises of identity time (Burke & Stets, 2009; Chen et al., 2023), it is anticipated that there be low levels of change across time for identity, compared to the other determinants of behaviour.

As most current evidence suggests a strong correlational relationship between identity and behaviour, it is hypothesised that (H1) identity will act as a behavioural determinant for each of the three behaviour types investigated in the current study; however, given the theory that identity is quite stable, it is hypothesised that these effects will be most reliable at the between-person level. Further, given that current research remains limited regarding whether the influence of identity on behaviour is direct or indirect through other motivational constructs, it is further hypothesised that (H2) the influence of identity on behaviour will wane or wash out entirely when accounting for intention, self-determined motivation, and habit.

## 2. Method

To investigate the influence of identity within- and between-person and across types of behaviours, the current research employed repeated self-reported assessments across time. Prior to its conduct, this study received full ethical approval from the Central Queensland University Human Research Ethics Committee (approval number 0000023907).

### 2.1. Participants and Recruitment

Participants were initially recruited via snowball social media postings and university class announcement forums. Participant eligibility included persons aged > 14 years, with the ability to provide informed consent and willingness to complete the 12 repeated assessment surveys. The broad age range was deemed appropriate to capture an inclusive representation of identity development stages (Marcia, 1993). Participants also needed to be residents of Australia, given that participant incentives were only approved for use in Australia.

As the research questions were at the within-person level and were to be tested within multilevel models, power is impacted by both *N* (sample size) and *n* (number of assessments; Arend & Schäfer, 2019). To be powered at 0.80 for the anticipated medium effect size, a minimum detectable effect size of 0.22, an *N* of 30 (participants) and an *n* of 12 (repeated measures) was needed and achieved.

### 2.2. Procedure

Once participants clicked the hyperlink in the participation invitation, they were taken to an online survey platform (Qualtrics, 2005). Participants were presented with study information before providing informed consent. Parents of participants < 16 years were emailed to notify them of their child’s provision of informed consent and given the opportunity to respond to that email with any questions or concerns. Those who opted to provide informed consent were immediately directed to the first of 12 weekly surveys, to self-report basic demographic items, behavioural frequency, identity, intention, self-determined motivation and habit measures. Open-ended qualitative questions pertaining to perceptions of identity were also asked in the initial, week 6, and week 12 surveys. These items were included within the survey to understand potential changes in perception of identity over time; however, as identity has been previously reported as a relatively stable concept (Chen et al., 2023; Cushan-Kain et al., 2022), we considered weekly inclusion of these items to be an unnecessary burden for participants. These items are not analysed nor presented within this study. Participants received consecutive weekly emails containing links to the weekly surveys. Participants were asked to complete each survey within 24 hours of receiving the link. The subsequent 11 weekly surveys were replicates of the initial survey, excluding the demographic items. On completion of survey six, participants were emailed an AUD 10 Prezzee gift voucher to incentivise their continued participation. At the completion of survey 12, participants were emailed an AUD 15 Prezzee gift voucher as final remuneration.

### 2.3. Measures

The full initial survey is publicly available via the Open Science Framework (OSF; https://osf.io/kd8n6/) (accessed on 23 March 2025).

#### 2.3.1. Participant Demographics

Participants self-reported personal demographics such as their age (open numerical response), gender (male; female; non-binary; I use a different term [please specify]), current relationship status (single; partnered in a relationship but not living together); married or de facto; separated or divorced; widowed; different relationship status [please specify]), and ethnicity (multiple responses allowed; Caucasian, Aboriginal; Torres Strait Islander; Asian; Pacific Islander; African; European; I belong to a different ethnic group [please specify]). Participants also self-reported their current work engagement (multiple responses allowed; full-time employee; part-time or casual employee; stay-at-home parent or carer; regular voluntary work; unemployed or between jobs; retired or unable to work; my work status is something different [please specify]), and education engagement (multiple responses allowed; studying at high school [secondary school]; studying at TAFE [trade college or apprenticeship]; studying at university [higher education degree]; I am studying somewhere different [please specify]; I am not currently studying).

#### 2.3.2. Behavioural Engagement

At the top of each survey page, a brief description of the three behaviours was provided for participants: “In this study, ‘physical activities’ are defined as activities that take physical effort and make you breathe harder than normal (e.g., heavy lifting, digging, aerobics, jogging/running, etc.)”; “In this study, ‘student behaviours’ are defined as engagements in any education or training that you are currently undertaking at home, online, in-class, or on-the-job (e.g., lectures/tutorials/workshops, assigned readings/tasks, graded assessments, etc.)”; and “In this study, ‘support seeking’ is defined as seeking out and engaging with supports classified as professional (e.g., counsellors, doctors, supervisors) and/or non-professional (e.g., family or friends)”.

Behavioural engagement for physical activity, student behaviour, and support seeking was self-reported via adaptions of the International Physical Activity Questionnaire (IPAQ-SF; Craig et al., 2003). The IPAQ-SF is a validated and reliable measure commonly used in exercise and physical activity research (Craig et al., 2003; Lee et al., 2011; van Poppel et al., 2010). Adaptations of IPAQ-SF questions were presented for each of the three behaviour types; for example, “During the last 7 days, on how many days did you engage in physical activities [or student behaviours or support seeking behaviour] (as described above)?” and “How much time did you usually spend engaging in physical activities [or student behaviours or support seeking behaviour] on any one of those days? [response in hours, minutes]”. As per validated scoring procedures (IPAQ Group, 2005), the total time of behaviour for the previous seven days was calculated to create a variable of total behaviour minutes for each independent behaviour, for each participant, at each timepoint.

#### 2.3.3. Identity

Identity relevant to each of the behaviour types (physical activity identity, student identity, support-seeking identity) was measured via adaptions of the Exercise Identity Scale (EIS; Anderson & Cychosz, 1994). The EIS is the most common validated and reliable measure of role identity and exercise beliefs and has also been used in variations to measure a variety of behavioural identities (Smith et al., 2008; Sparks & Shepherd, 1992; Wilson & Muon, 2008). Adaptations of questions were created by replacing ‘exercise’ wording with the relevant behaviour wording; for example, “I consider myself a physically active person”, “I consider myself a student”, and “I consider myself a person willing to seek support” (for further examples of adaptations, please find the full survey available in Supplementary Materials via https://osf.io/kd8n6/) (accessed on 23 March 2025). Responses for each item were based on a seven-point Likert scale (1 strongly disagree–7 strongly agree). The nine EIS items were averaged to produce a single identity score for each behaviour, for each participant, at each timepoint, with higher scores representing stronger identification for the relevant identity. Interitem reliability for the EIS in the current study (α = 0.94) reveals strong internal consistency.

#### 2.3.4. Intention Strength

Behavioural intentions for each behaviour were measured as adaptions of intention strength items as presented in Rhodes and Rebar (2017). Participants were asked: “Over the next 7 days… TO WHAT EXTENT do you intend to engage in physical activities [or student behaviours or support seeking behaviours]?”. Responses for each item were based on a seven-point Likert scale (1 not at all–7 a lot), resulting in a single item score of intention strength for each behaviour, for each participant, at each timepoint, with higher scores reflective of stronger intention for the relative behaviour.

#### 2.3.5. Self-Determined Motivation

Self-determined motivation for each of the behaviours was measured via adapted subsets of items from the Behavioural Regulation for Exercise Questionnaire (BREQ-3; Markland & Tobin, 2004). For this study, two items from each of the external, introjected, identified, integrated, and intrinsic regulation subsets were used to maintain a shorter length of survey while also capturing data for each level of behavioural regulation. Examples of behaviour-relevant adaptations include “I feel guilty when I’m not physically active”, “I engage in student behaviours because other people say I should”, and “I value the benefits of seeking support” (for further examples of adaptations, please find the full survey available in Supplementary Materials via https://osf.io/kd8n6/). Response options were a five-point scale (0 not true for me–4 very true for me). The responses were scored to produce a relative autonomy index (RAI; Ryan & Connell, 1989), a unidimensional index of the degree of self-determination, for each behaviour, for each participant, at each timepoint. Higher RAI scores being reflective of stronger self-determined motivation for the relevant behaviour.

#### 2.3.6. Habit Strength

Behavioural habit strength for each behaviour was measured via the Self-Reported Behavioural Automaticity Index (SRBAI; Gardner et al., 2012a). The SRBAI is a validated and reliable automaticity subscale of the Self-Report Habit Index (Verplanken & Orbell, 2003). The SRBAI question stems included “Being physically active is something you…”, “Student behaviours are something you…”, and “Support seeking is something you…” and were followed by “do automatically”, “do without having to consciously remember”, “do without thinking”, and “start doing before you realise you’re doing it”. Response options were seven-point scales (1 strongly disagree–7 strongly agree). The four SRBAI items for each behaviour were averaged to create a single item relative for each behaviour, for each participant, at each timepoint, with higher scores representing stronger habit for the relative behaviour. Interitem reliability for the SRBAI in the current study was strong (α = 0.96).

### 2.4. Data Management and Analyses

Data management and analyses were conducted in R (version 2024.09.0+375; R Core Team, 2019). Ninety-eight participants completed the initial survey. However, across all 12 surveys, the total attrition rate was 72.5%. Of the 98 participants who completed the initial survey, 66 participants (67%) continued participation to complete the first 3 of the 12 weekly surveys (25%), 45 participants (46%) continued to complete 6 of the 12 surveys (50%), 33 participants (34%) continued to complete 9 of the 12 surveys (75%), and 27 participants (28%) continued to complete all 12 of the 12 weekly surveys (100%). Despite the attrition rate, statistical power was maintained, based on repeated measures parameters presented by Arend and Schäfer (2019).

Intraclass correlations were run with the R ICC package (version 2.4.0; Wolak et al., 2012) to determine the ratio of between-person to within-person variability of each variable. Assumption testing was further conducted for multilevel modelling (e.g., normality, linearity, homoscedasticity; version 1.1-7; Bates, 2014). All three behaviour variables (physical activity, student behaviour, support seeking) violated assumptions of normality and were subsequently truncated to the 75% interquartile values to reduce the risk of undue influence from outliers. Histograms of variables (pre- and post-truncation) along with the complete R script are presented within Supplementary Materials (available via OSF https://osf.io/kd8n6/) (accessed on 23 March 2025).

Multilevel regression models were run with the R lmer package (Bates, 2014), separately for each behavioural dataset in a stepwise manner. Prior to analysis, identity variables were parsed to create between- (means-centred) and within-person (residuals) level variables. The first step of the models was behaviour regressed onto between- and within-person identity; the second step of the models was behaviour regressed onto between- and within-person identity and intention; the third step was behaviour regressed onto between- and within-person identity and self-determined motivation; and the final step was behaviour regressed onto between- and within-person identity and habit.

## 3. Results

### 3.1. Sample Characteristics

Sample characteristics are presented in Table 1. Ninety-eight participants were recruited (*M* age = 30.4 years, *SD* = 11.7 years; 80.6% female, 14.3% male, 5.1% non-binary/fluid/other gender), and all participant responses were included in analyses to facilitate statistical power (as per Arend & Schäfer, 2019). Most participants (51.0%) were married or in a de facto relationship and reported Caucasian ethnicity (79.6%). More than half of the participants (51.0%) described their work status as part-time or casual, and 85.7% of participants reported that they were studying at tertiary level (university). 

### 3.2. Study Descriptives

Person-level descriptive statistics, intraclass correlations, and bivariate correlations for the three behaviours are shown in Table 2. Participants engaged in physical activity for more than twice the recommended weekly minutes of 150 min (Department of Health and Aged Care, 2021). On average, student behaviour minutes matched those typical of part-time study loads, while support seeking behaviour minutes were the lowest among the three behaviours. Participants scored their identity related to physical activity and student behaviour moderately near the mid-point on the scale, but lower for support seeking. Intention strength was highest for student behaviour, followed by physical activity and support seeking. Habit strength was generally low across all behaviours, with student behaviour and physical activity only slightly higher than support seeking, Self-determined motivation was moderate for physical activity and student behaviour, but much lower for support seeking. Intraclass correlations revealed that about one-third of the variability in behaviours was at the between-person level, whereas for all behaviours, identity and habit were strongly stable with most variability at the between-person level. Intention strength tended to be nearly equally split between variability at the between- and within-person levels. In terms of bivariate correlations, given that the nesting in the data is not accounted for in correlations, no statistical significance testing was conducted. In all, the bivariate correlation results suggest identity for each of the three behaviours is most strongly related to self-determined motivation, followed by intention and habit; and least related to time spent engaging in the relevant behaviour (mins/week).

### 3.3. Stepwise Multilevel Models

#### 3.3.1. Physical Activity

As shown in Table 3, when tested as an independent predictor, identity was found to be statistically and significantly, positively associated with physical activity behaviour at both the between- and within-person levels. This result suggests people with a stronger physical activity identity were likely to engage in more physical activity than people with a weaker physical activity identity; and also that, on weeks when the person’s physical activity identity was stronger, they were likely to engage in more physical activity than on weeks when their physical activity identity was weaker.

Intention strength was significantly associated with physical activity behaviour, and the relationship between physical activity identity and behaviour remained statistically significant after controlling for intention at the between-person level. However, at the within-person level, the relationship between identity and behaviour did not remain significant when accounting for intention. This finding suggests that once the shared variability in intention and within-person fluctuations in physical activity identity were accounted for, the link between physical activity identity and behaviour was mitigated. Self-determined motivation as an independent predictor of physical activity was non-significant. When accounting for self-determined motivation, both within- and between-person identity remained statistically significantly, positively associated with physical activity. Habit was significantly associated with physical activity behaviour; suggesting that habit was an independent predictor of physical activity. When accounting for habit, the positive associations between both within- and between-person identity and physical activity behaviour remained statistically significant. Taken together, these findings suggest that the relationship between physical activity identity and physical activity behaviour was present at both between- and within-person levels, even after accounting for intention, self-determined motivation and habit, with the exception that the influence of within-person fluctuations of physical activity identity on behaviour was at least partially shared with intention.

#### 3.3.2. Student Behaviour

As shown in Table 4, student identity significantly and positively predicted student behaviour at both the within-person and between-person levels. This result suggests people with a stronger student identity were likely to engage in more student behaviours than people with a weaker student identity; and also that, on weeks when the person’s student identity was stronger, they were likely to engage in more student behaviour than on weeks when their student identity was weaker.

Intention strength was significantly associated with student behaviour. The relationships between student identity and student behaviour did not remain significant when controlling for intention, at either the between- and within-person level, suggesting that identity and intention have a shared effect on student behaviour. Self-determined motivation was negatively, but not significantly, associated with student behaviour. However, the relationship between student identity and student behaviour remained positive and significant when controlling for self-determined motivation, at both the between- and within-person level. Habit was significantly associated with student behaviour; and the link between student identity and student behaviour remained significant when accounting for habit, at both the between- and within-person level. Together, the results suggest that the relationship between student identity and student behaviour is at both the between-person and within-person levels, even after accounting for self-determined motivation and habit. However, the influence of both within- and between-person student identity was shared with student behaviour intention strength.

#### 3.3.3. Support Seeking

As shown in Table 5, when tested as an independent predictor, support-seeking identity was found to be significantly, positively associated with support-seeking behaviour at the between-person level, but not at the within-person level. This result suggests people with a stronger support-seeking identity were likely to engage in more support-seeking behaviour compared to those with a weaker support-seeking identity; but that fluctuations in a person’s support-seeking identity week-to-week did not mean they engaged in more or less support-seeking behaviour in a given week.

Intention strength was statistically significantly associated with support-seeking behaviour, and the relationship between support-seeking identity and behaviour remained statistically significant after controlling for intention at the between-person level. The association between intention and support-seeking behaviour was not impacted by the inclusion of intention within the model. The relationship between support-seeking behaviour and self-determined motivation was non-significant and when accounting for self-determined motivation, the relationship between support-seeking identity and support-seeking behaviour remained significant at the between-person level. This suggests that self-determined motivation did not predict support-seeking behaviour and did not impact the identity–behaviour association. Habit was statistically significantly, positively associated with support-seeking behaviour and accounting for habit did not impact the relationship between support-seeking motivation and support-seeking behaviour. Altogether, these findings suggest that the relationship between support-seeking identity and behaviour was at the between-person level and that this relationship did not change after accounting for intention, self-determined motivation or habit.

## 4. Discussion

To expand the existing evidence regarding identity–behaviour relationships and move towards understanding how and when identity may be integrated within behaviour change processes, the current study investigated the influence of identity across three behaviour types. Findings from the current study that most variability in identity was at the between-person level highlights the stability of identity over time. Identity was associated with physical activity behaviour and student behaviour at both the between- and within-person level, but only at the between-person level for support seeking (supporting H1). However, for physical activity behaviour and student behaviour, the relationship between identity and behaviour became non-significant when accounting for intention, but not when accounting for self-determined motivation or habit (supporting H2), suggesting there may be some shared influence between identity and intention.

### 4.1. The Influence of Identity Across Behaviours

Identity was found to be largely stable across time and was associated with all three distinct behaviours, physical activity, student behaviour, and supporting seeking, at (at least) the between-person level. The ICCs of the study revealed that most of the variability in identity was a result of individual differences rather than within-person change over time and context. The stability of identity and its impact on behaviour found in this study aligns with the results of previous identity–habit research (Cushan-Kain et al., 2022) and supports the conceptualisations of identity as a stable construct (Chen et al., 2023).

That within-person weekly fluctuations in identity for support seeking were not associated with behavioural engagement in support seeking is likely the by-product of the low variability left to predict behaviour at the within-person level. However, notably, there were within-person significant associations for identity and physical activity and student behaviours. This suggests that, for some circumstances, fluctuations in identity may be meaningful for influencing behaviour. It may be that fluctuations in identity are reflective of the salience of identity for the relevant behaviour. That is, rather than identity shifting week-to-week, it may be that identity becomes more or less relevant and conscious depending on the dynamics of context and behavioural engagement (Cushan-Kain et al., 2022; West & Brown, 2013). So, our findings may be interpretable as on weeks when behavioural identity was more salient, the behaviour was engaged in more often.

That the within-person association between identity and behaviour did not play out for support-seeking behaviour may also be due to the nature of how social support is engaged in. Unlike physical activity or student behaviour, support seeking involves the management of perceived or real stigma and social expectations (Corrigan & Rao, 2012; Gardner et al., 2018; Klinner et al., 2023; Rüsch et al., 2005). Support seeking involves overcoming barriers that are not as apparent for physical activity and student behaviour. For example, a person is likely to encourage another to seek support but may be hesitant to seek support themselves (Gardner et al., 2018; Klinner et al., 2023; Myers et al., 2012). There may also be a real or perceived lack of access to support; there may be limited social or professional support systems, as often seen in rural and remote communities (Klinner et al., 2023), or there may be support opportunities but the individual may not have the self-awareness or efficacy to pursue those supports (Corrigan & Rao, 2012). So, it may be that identity for support seeking becomes salient one week, but fear of stigma or lack of access interferes with engaging in the behaviour. Given these additional obstacles to support-seeking behaviour, consideration of support-seeking identity may be less warranted when developing interventions for support-seeking behaviour. Rather, interventions that promote factors such as empowerment, self-efficacy and stigma reduction may be more beneficial for improving support-seeking behaviour (Corrigan & Rao, 2012; Rüsch et al., 2005).

### 4.2. The Shared Influence of Identity and Intention

Intention strength was significantly associated with all three behaviours, supporting the plethora of evidence suggesting that intention strength is a primary determinant of behaviour (Ajzen, 1985; Ajzen & Madden, 1986; Bagozzi, 1992; Rhodes & Rebar, 2017; Sheeran, 2002). Notably, the current results showed the relationship between physical activity and identity, and student behaviour and identity, waned when accounting for intention, but not self-determined motivation or habit. These findings suggest that intention and identity work through the same mechanisms, or that they interact with each other, to influence behaviour. Given the current findings alongside existing literature which suggests the influence of identity is important to the formation of behavioural intention (Ajzen, 1991; Berry et al., 2014; Fielding et al., 2008; Fishbein & Ajzen, 2010; Gardner & Lally, 2013; Hagger & Chatzisarantis, 2006), we may speculate that identity works to strengthen commitment to behavioural intention through salience of values relevant to the behaviour. Due to lacking statistical power, the current study did not test the mediating or moderating effects of this identity–intention relationship and thereby further research is needed to clarify the nature of this association.

Self-determined motivation was, surprisingly, not significantly associated with any of the three behaviours. This finding contradicts the strong evidence suggesting self-determined motivation as a primary predictor of behaviour (Deci & Ryan, 1980, 2002; Gardner & Lally, 2013; Mullan & Markland, 1997; Ryan & Deci, 2000; Teixeira et al., 2012). It may be considered, however, that the behaviours in this study are unlikely to elicit inherent intrinsic value; for example, moderate physical activity is less enjoyable compared to vigorous physical activity (Hsu et al., 2022; Thum et al., 2017) and student engagements measured during pandemic-related educational reform reflect reduced enjoyment (Hari Rajan et al., 2024). It also may be that the studied behaviours are simply a means to an end (e.g., engaging in student behaviour leads to a certificate or degree, which may lead to job attainment/promotion; support seeking leads to support attainment, which ideally leads to improved mental wellbeing), and thereby less intrinsically valuable. An association between identity and behaviour was apparent both between- and within-person after accounting for self-determined motivation for all behaviours, except support seeking where the identity–behaviour relationship remained non-significant at the within-person level. These findings suggest that identity influences behaviour independently from the effects of self-determined motivation.

Habit strength was also significantly associated with all three behaviours in the current study. These results align with the vast evidence suggesting habit is a primary determinant of behaviour (Gardner, 2015; Gardner et al., 2011, 2024; Gardner & Rebar, 2019; Lally et al., 2010). The link between identity and behaviour remained after accounting for habit, for all three behaviours, suggesting that habit is likely to work independently from identity to influence behaviour. As such, interventions that seek to promote self-determined motivation and habit formation may be further strengthened by the inclusion of mechanisms that align identity and the desired behaviour. For example, interventions that seek to improve student retention and engagement may consider promoting alignment of student identity with positive study-related outcomes (Chorba et al., 2012; Hardy et al., 2013).

Altogether, the current results suggest that identity may influence different behaviours in different ways (e.g., between- and/or within-person). Results also suggest that identity influences behaviour independently from self-determined motivation and habit, but through, or in the same way as, intention. These results may help to explain the inconsistent findings of previous identity–behaviour research. By considering the dynamic within- and between-persons effects, different behaviour types, and key behavioural determinants of behaviour change within a singular study, the current study presents a level of conceptual, measurement, and outcome consistency that previous research has not offered. With the consistency offered by the current study, it can be suggested that the variability across previous research findings is due to conceptualisation and measurement factors. It may also be suggested that the variability is due to how the identity–behaviour relationship is influenced by factors such as social, cultural and personal differences, and identity’s relationship with other behavioural determinants across the stages of behaviour change. From the current study, future research is encouraged to delve deeper into the potential theoretical and practical caveats that may be influential on the relationship between identity and behaviour.

### 4.3. Implications and Future Directions

Many behavioural interventions containing identity components currently rely on participants’ reflection of their identity standards and role engagement (Barnett et al., 2021; Berkman et al., 2017; Burke, 2006). Reflecting on, for example, exercise identity to increase exercise behaviour may be effective when the individual is able to reflect on how their exercise identity aligns with their recent exercise behaviours. However, this assumes the individual has completed the intention formation stage, successfully managed the intention–behaviour gap, and is in the process of the action initiation stage of behaviour change (Heckhausen & Gollwitzer, 1987; Heckhausen, 2007). Based on the current findings, reflections on how identity and a desired behaviour may align may be best implemented at the intention formation stage, to support the considerations pertaining to behavioural value and relevance versus risk assessment. Aligning values, identity, and behaviour at this stage of behaviour change may help to strengthen the commitment to the intention to engage in the behaviour, thereby potentially reducing the intention-behaviour gap and improving the likeliness of behavioural engagement (Berkman et al., 2017).

The current findings support the identity–behaviour relationship and that identity is stable over time. Given the stability of identity, it may be considered that behavioural interventions that seek to ‘change’ identity have limited effectiveness (Barnett et al., 2021). Rather, identity-related behavioural interventions may seek to tailor interventions that align identity with behaviours that reach the desired outcome. For example, a person with a weak exercise identity but a strong health identity may be more inclined to align with and commit to a behaviour that supports health without requiring high levels of exercise (e.g., diet or lifestyle physical activity). By gradually introducing ‘exercise’ into the intervention, there may be potential for the person to integrate ‘exercise’ into their health identity. Interventions that target other motivational precursors, such as efficacy, social support, and autonomy, may indirectly influence identity by enhancing behavioural engagement (Judge et al., 2022; Manninen et al., 2022). While the idea of identity shifts has been evidenced in the literature (Adams & Serpe, 2020; Burke, 2006), further research is required to substantiate the potential for identity change across different behavioural contexts.

### 4.4. Study Limitations

Our repeated measure approach required participants to reflect on their daily behaviours and motivation, and it should be acknowledged that the subjectivity in this reflection may provide inaccurate accounts of daily experiences (Gardner et al., 2012a; Hagger, 2016; Rebar et al., 2016; Sniehotta & Presseau, 2011), particularly for variables suggested as processed outside of reflective consciousness, such as identity (Rhodes, 2017). Obtaining device- or passively assessed measures alongside self-report is a common approach to mitigate this bias; future identity–behaviour research is encouraged to utilise these such approaches.

While statistical power was achieved through the repeated measures design (Arend & Schäfer, 2019), a second limitation is noted in terms of the high participant attrition. Despite designing the study in a way that reduced participant fatigue and potential inconveniences, while maintaining an appropriate and effective survey structure, the study experienced very high attrition across the 12 weeks of the survey. Future research may seek to replicate the current study by collecting data across shorter data collection intervals or with a more focused population sample more likely to maintain their study participation (via interest or incentive). Also, while not the aim of the current study, future research may aim for appropriate statistical power to include moderation or mediation models to test the nature of the shared variability between identity, intention, and behaviour.

It should also be noted that participants in this study reported a strong identity for physical activity and a very high weekly engagement in physical activity, more than double the recommended Australian guidelines of around 150 minutes per week (Department of Health and Aged Care, 2021). Reported student identity was also strong, and student behaviour was reflective of part-time educational engagement, potentially reflecting the work-study duality of the mid-adult age sample. Support-seeking identity and behaviour were the lowest reported identity and behaviour, potentially reflecting the stigma generally attached to mental health and wellbeing within many Australian populations (Gardner et al., 2018; Klinner et al., 2023), and student populations (Myers et al., 2012). As such, it may be considered that the current results are representative of a highly physically active student population. However, as most participants reported being mid-adult age with a part-time student load, results may also be translatable to a broader population of working adults. In any case, results may only be interpreted in the context of the studied behaviours, behavioural mechanisms, and identity types. While these findings may align with other behaviours and identity types and provide valuable insight and clarification for the identity–behaviour field, further investigation of various behaviour types, behavioural mechanisms, identity types, and populations is encouraged to expand upon the current findings.

Finally, given that the current results highlight the variability in how identity influences different behaviour types and behavioural determinants, future behaviour research may need to consider theoretical and conceptual refinement to clearly convey the type of behaviour being studied, the relevant behavioural mechanisms for that behaviour and behaviour change process, and how the variables of interest are being measured. For example, research investigating how an intention for a specific behaviour may be influenced by social identity should consider how the theoretical conceptualisation of intention may be interpreted alongside the theoretical conventions of social identity. Future research should also consider additional relevant behavioural determinants that may influence the identity–behaviour relationship. For example, self-control (Hagger & Hamilton, 2024), self-esteem (Pazzaglia et al., 2020), self-regulation techniques (Nurmi et al., 2016), and autonomy support (Fenton et al., 2014) are evidenced to influence behaviour and align with the self and social concepts featured within identity theories (Burke, 1980; Stets & Burke, 2000; Stryker, 1968). The inclusion of such determinants may help to further explain how and when identity may be likely to influence behaviour. To echo previous calls (e.g., Alfrey et al., 2023; García et al., 2023; Kindelberger et al., 2020; Murnieks et al., 2014), future research must move beyond correlational design to investigate the deeper theoretical and environmental intricacies and dynamics that underlie both identity (e.g., identity type) and behaviour models (stage of behaviour change and/or behaviour type and/or behavioural determinants).

## 5. Conclusions

Existing research has highlighted the relationship between identity and behaviour; however, evidence reveals inconsistent findings regarding how identity may influence behaviour. The current study adds evidence by studying identity–behaviour relationships consistently across behaviour types (physical activity, student behaviour, support seeking) and behavioural mechanisms (intention, motivation, habit). Findings reinforce the notion of identity having a direct influence on behaviour, predominantly at the between-person level, and suggest identity as largely stable within-person, over time. Physical activity and student behaviour were associated with identity at both the between- and within-person level; however, support seeking was only associated with identity at the between-person level, suggesting that fluctuations in support seeking may be influenced by mechanisms other than identity. Findings further highlight that identity may influence physical activity and student behaviour independently from self-determined motivation and habit, but through or in the same ways as intention. While identity has evidenced influence on behaviour, the empirical inconsistencies regarding how and when identity’s influence may be appropriate for behaviour change have resulted in uncertainties regarding the integration of identity into behavioural interventions. Findings from the current study suggest that, due to its stability over time, identity may be difficult to change. However, tailoring the alignment of identity and behaviour during the intention formation stage of behaviour change may help to increase intention strength, mitigate the intention-behaviour gap, and improve the likelihood of successful behaviour change.

## Figures and Tables

**Table 1:** Participant characteristics (*n* = 98).

Participant Characteristic		Frequency (*n*)	Frequency (%)
Gender	Female	79	80.6
	Male	14	14.3
	Non-binary, fluid, or other gender	5	5.1
Relationship Status	Married/De facto	50	51.0
	Single	26	26.5
	Partnered	18	18.4
	Separated/Divorced	4	4.1
Ethnicity	Caucasian	78	79.6
	Mixed ethnicity	11	11.2
	European	5	5.1
	Asian	3	3.1
	Aboriginal	1	1.0
Work Status	Part-time/Casual	50	51.0
	Full-time	24	24.5
	Unemployed/Between jobs	15	15.3
	Stay-at-home duties	5	5.1
	Retired/Unable to work	3	3.1
	Voluntary duties	1	1.0
Education Status	Tertiary (University)	84	85.7
	Secondary study (high school)	7	7.2
	Not currently studying	6	6.1
	Tertiary (TAFE/Apprenticeship)	1	1.0

**Table 2:** Person-level descriptives, intraclass correlations, and bivariate correlations for behaviour, intention, motivation, and habit across physical activity, student behaviour and support-seeking behaviours.

		Descriptives	Intraclass Correlations	Bivariate Correlations			
		*M* (*SD*)	ICC (95% CI)	*r*			
				Identity	Intention	Motivation	Habit
Physical Activity							
	Behaviour ^†^	318.42 (463.00)	0.39 (0.30 to 0.49)	0.28	0.30	0.19	0.17
	Truncated to 75%	212.45 (160.07)					
	Identity	4.05 (1.56)	0.90 (0.87 to 0.93)	-	0.67	0.70	0.53
	Intention Strength	4.50 (1.54)	0.58 (0.50 to 0.67)		-	0.55	0.47
	Self-determined Motivation	9.99 (6.57)	0.88 (0.85 to 0.91)			-	0.44
	Habit Strength	3.25 (1.65)	0.75 (0.69 to 0.81)				-
Student Behaviour							
	Behaviour ^†^	1575.98 (2332.33)	0.39 (0.30 to 0.49)	0.14	0.32	0.04	0.15
	Truncated to 75%	1004.19 (740.64)					
	Identity	5.04 (1.40)	0.86 (0.82 to 0.90)	-	0.51	0.60	0.59
	Intention strength	5.18 (1.89)	0.40 (0.31 to 0.50)		-	0.31	0.33
	Self-determined Motivation	10.68 (5.74)	0.83 (0.79 to 0.87)			-	0.44
	Habit Strength	3.71 (1.79)	0.78 (0.72 to 0.83)				-
Support Seeking							
	Behaviour ^†^	84.55 (175.18)	0.32 (0.23 to 0.42)	0.46	0.37	0.30	0.37
	Truncated to 75%	40.51 (44.50)					
	Identity	3.18 (1.49)	0.83 (0.78 to 0.87)	-	0.68	0.73	0.70
	Intention Strength	2.55 (1.62)	0.66 (0.58 to 0.74)		-	0.59	0.61
	Self-determined Motivation	5.44 (6.07)	0.82 (0.78 to 0.87)			-	0.61
	Habit Strength	2.91 (1.80)	0.79 (0.73 to 0.84)				-

Note: *M* = Mean; *SD* = Standard Deviation; ICC = Intraclass Correlation; CI = Confidence Interval; *r* = Pearsons *r*; ^†^ Behaviour in weekly minutes.

**Table 3:** Stepwise multilevel model testing the association of between and within-person identity on physical activity behaviour, and after accounting for intention, self-determined motivation, and habit.

		Physical Activity Models			
		Coefficient	Standard Error	t-Statistic	95% CI
Between- and Within-person Identity					
	Intercept	18.597	32.792	0.567	−45.735 to 82.800
	Between-person Identity	47.479	7.598	6.249	32.611 to 62.400 *
	Within-person Identity	42.203	10.750	3.926	21.108 to 63.295 *
Between- and Within-person Identity + Intention Strength					
	Intercept	−30.346	32.752	−0.927	−94.358 to 33.789
	Between-person Identity	28.573	8.071	3.540	12.820 to 44.389 *
	Within-person Identity	21.110	11.433	1.846	−1.344 to 43.478
	Intention	27.601	5.136	5.374	17.578 to 37.717 *
Between- and Within-person Identity + Self-determined Motivation					
	Intercept	27.147	33.588	0.808	−38.462 to 92.775
	Between-person Identity	39.874	9.714	4.105	20.914 to 58.813 *
	Within-person Identity	39.993	10.880	3.676	18.677 to 61.341 *
	Self-determined Motivation	2.286	1.808	1.264	−1.251 to 5.811
Between- and Within-person Identity + Habit					
	Intercept	4.606	31.207	0.148	−56.383 to 65.615
	Between-person Identity	34.380	7.934	4.333	18.907 to 49.927 *
	Within-person Identity	33.050	10.961	3.015	11.518 to 54.498 *
	Habit	20.346	5.272	3.859	10.056 to 30.708 *

Note: CI = Confidence Interval; * Coefficients are statistically significantly different from zero with 95% confidence.

**Table 4:** Stepwise multilevel model testing the association of between and within-person identity on student behaviour, and after accounting for intention, self-determined motivation, and habit.

		Student Behaviour Models			
		Coefficient	Standard Error	t-Statistic	95% CI
Between- and Within-person Identity					
	Intercept	151.69	242.89	0.624	−324.881 to 627.085
	Between-person Identity	169.77	46.62	3.642	78.513 to 261.223 *
	Within-person Identity	144.36	49.60	2.911	47.055 to 241.655 *
Between- and Within-person Identity + Intention Strength					
	Intercept	−89.01	217.41	−0.409	−514.554 to 336.865
	Between-person Identity	59.18	42.81	1.382	−24.449 to 143.211
	Within-person Identity	67.96	46.70	1.455	−23.673 to 159.382
	Intention	152.43	14.94	10.202	123.248 to 182.153 *
Between- and Within-person Identity + Self-determined Motivation					
	Intercept	118.627	243.340	0.487	−357.278 to 593.983
	Between-person Identity	196.858	52.143	3.775	94.999 to 298.595 *
	Within-person Identity	151.844	50.058	3.033	53.764 to 250.030 *
	Self-determined Motivation	−9.889	8.695	−1.137	−26.926 to 7.068
Between- and Within-person Identity + Habit					
	Intercept	154.52	236.60	0.653	−309.180 to 616.497
	Between-person Identity	115.21	48.99	2.352	19.652 to 211.190 *
	Within-person Identity	120.30	50.08	2.402	22.072 to 218.393 *
	Habit	73.84	24.94	2.961	25.205 to 122.726 *

Note: CI = Confidence Interval; * Coefficients are statistically significantly different from zero with 95% confidence.

**Table 5:** Stepwise multilevel model testing the association of between and within-person identity on support-seeking behaviour, and after accounting for intention, self-determined motivation, and habit.

		Support-Seeking Behaviour Models			
		Coefficient	Standard Error	t-Statistic	95% CI
Between- and Within-person Identity					
	Intercept	−12.494	7.481	−1.670	−27.104 to 2.282
	Between-person Identity	17.157	2.063	8.316	13.089 to 21.186 *
	Within-person Identity	3.160	2.368	1.334	−1.490 to 7.805
Between- and Within-person Identity + Intention Strength					
	Intercept	−16.172	6.587	−2.455	−29.030 to −3.213 *
	Between-person Identity	9.020	2.074	4.349	4.969 to 13.068 *
	Within-person Identity	−1.355	2.298	−0.589	−5.874 to 3.140
	Intention	11.173	1.253	8.919	8.729 to 13.638 *
Between- and Within-person Identity + Self-determined Motivation					
	Intercept	−9.517	7.718	−1.233	−24.563 to 5.627
	Between-person Identity	15.073	2.514	5.997	10.167 to 19.972 *
	Within-person Identity	2.162	2.468	0.876	−2.683 to 6.991
	Self-determined Motivation	0.692	0.481	1.439	−0.245 to 1.632
Between- and Within-person Identity + Habit					
	Intercept	−14.137	6.881	−2.055	−27.555 to −0.616 *
	Between-person Identity	10.611	2.253	4.710	6.216 to 15.004 *
	Within-person Identity	−0.728	2.434	−0.299	−5.507 to 4.034
	Habit	7.714	1.400	5.509	4.984 to 10.454 *

Note: CI = Confidence Interval; * Coefficients are statistically significantly different from zero with 95% confidence.

## Data Availability

Deidentified data may be made available upon reasonable request, by emailing the corresponding author (k.alfrey@cqu.edu.au).

## Supplementary Materials

Supplementary materials are available via the Open Science Framework (OSF; https://osf.io/kd8n6/) (accessed on 23 March 2025).
